# Pharmacists’ Work Experiences and Career Dynamics in Saudi Arabia: A Cross-Sector Study

**DOI:** 10.3390/pharmacy14010018

**Published:** 2026-01-27

**Authors:** Mohammed Alnuhait, Ayidh Alqarni, Leena Alsharafi, Arjwan Alshreef, Renad Althebaiti, Alaa Shahbar, Foud Bahamdain, Abdulhamid Althagafi, Mohamed A. Albekery, Abdullah F. Alharthi, Abdulmalik S. Alotaibi

**Affiliations:** 1 Department of Clinical Pharmacy, College of Pharmacy, Shaqra University, Al-Dawadmi Campus, Al-Dawadmi 11961, Saudi Arabia; 2 Pharmaceutical Practices Department, College of Pharmacy, Umm Al-Qura University, Makkah 24381, Saudi Arabia; 3 Department of Pharmaceutical Care Services, King Abdullah Medical City, Makkah 24246, Saudi Arabia; 4 Department of Pharmacy Practice, Faculty of Pharmacy, King Abdulaziz University, Jeddah 22252, Saudi Arabia; 5 Department of Pharmacy Practice, College of Clinical Pharmacy, King Faisal University, Al-Ahsa 31982, Saudi Arabia

**Keywords:** Saudi Arabia, pharmacists, job satisfaction, burnout, well-being, workplace environment, career mobility, professional development

## Abstract

Background: Pharmacists in Saudi Arabia are assuming increasingly diverse and specialized roles amid rapid healthcare transformation. However, evolving expectations and expanding responsibilities may influence their job satisfaction, well-being, and career stability. This study aimed to assess job satisfaction, burnout, well-being, and career intentions among pharmacists across multiple practice sectors in Saudi Arabia. Method: A nationwide cross-sectional survey was conducted between December 2024 and January 2025 using an electronic questionnaire distributed to licensed pharmacists. The instrument assessed mental well-being, job satisfaction, burnout, workplace environment, and career mobility. Descriptive and inferential analyses were performed using SPSS version 20.0. Results: A total of 531 pharmacists completed the survey; 65% were male, and 89.3% were Saudi nationals. Sector distribution differed significantly by gender (*p* < 0.001): females were more represented in clinical and hospital pharmacy, while males predominated in the pharmaceutical industry–related roles. Male pharmacists reported higher work environment scores (*p* = 0.028) and greater sector mobility (34.2% vs. 23.7%, *p* = 0.012). Approximately 30.5% of participants had changed their employment sector at least once. Community pharmacists reported the highest burnout levels, whereas those in regulatory and administrative roles demonstrated the greatest job satisfaction (both *p* < 0.001). Participation in professional development showed strong positive associations with job satisfaction and intention to remain in the current role. Conclusions: Marked variations exist in pharmacists’ well-being, satisfaction, and career mobility across sectors in Saudi Arabia, with notable gender differences. Enhancing professional development, ensuring equitable work environments, and promoting sector-specific support strategies may help inform discussions on pharmacist engagement and retention within the evolving national healthcare system.

## 1. Introduction

Pharmacists in Saudi Arabia have witnessed expanding roles and evolving responsibilities in recent years, mainly driven by the country’s healthcare transformation programs focused on workforce development and the delivery of healthcare. Saudi Arabia’s Vision 2030 establishes a comprehensive strategic framework to revitalize the national economy and social sectors, including healthcare, through extensive changes designed to enhance services, augment worker capacity, and elevate the efficiency and quality of treatment throughout the Kingdom. While numerous workforce studies have investigated general health labor markets and identified shortages and distribution challenges among professions like physicians and nurses, substantial gaps exist in research specifically focusing on pharmacist workforce dynamics [1,2].

Pharmacists across different practice sectors face substantial workplace challenges, particularly related to job satisfaction, well-being, and career stability. Recent evidence highlights notable variations in burnout, satisfaction levels, and career intentions among pharmacists across sectors, such as hospitals, community chain pharmacies, community independent pharmacies, primary care center pharmacies, industrial pharmacies, and academia. Clinical pharmacists typically practice in hospital-based or ambulatory care settings, where they provide direct patient-centered services such as medication therapy optimization, therapeutic monitoring, and participation in multidisciplinary clinical teams, often within specialized services [3,4]. Among these, community pharmacists appear to be the most affected by burnout, with nearly 83.6% reporting personal burnout and 76.1% experiencing client-related burnout, often linked to extended working hours and heavy dispensing responsibilities. In addition to workload-related stress, income and salary dissatisfaction have been consistently reported as major factors influencing recruitment challenges and decisions to leave community pharmacy practice [3]. Even in hospital settings, where the nature of work is more clinical, emotional exhaustion has been reported in about 42.7% of pharmacists, exacerbated by role ambiguity and challenges in interdisciplinary collaboration [4,5,6]. Conversely, pharmacists working in the pharmaceutical industry, particularly medical representatives, report notably higher levels of job satisfaction. This has been attributed to competitive salaries, structured career development opportunities, and perceived social impact [4,5,6,7]. Gender-related challenges further complicate the pharmacy workforce landscape. Female pharmacists, particularly in community practice, face higher attrition rates, nearly 3.2 times greater than their male counterparts, often due to misaligned workplace cultures and limited leadership opportunities [8,9]. This is consistent with data showing that only 17% of female pharmacy students preferred community pharmacy as a future career, despite this sector providing the majority (68%) of pharmacy jobs in the country [9]. The COVID-19 pandemic added another layer of stress across sectors, with 33.7% of pharmacists reporting deterioration in mental health and 53% experiencing accelerated emotional exhaustion, especially in public hospitals [10,11,12]. Beyond pandemic effects, ongoing challenges such as financial pressures, workload intensity, and limited clinical autonomy continue to drive dissatisfaction, particularly in community settings, where only 48% report job satisfaction compared to 63.4% in hospitals [4,13]. Work environment factors, including workload and organizational support, have been strongly associated with satisfaction and burnout levels. Studies report significant negative correlations between workload intensity and satisfaction, and occupational stress has been shown to reduce job satisfaction [4,14]. Career intentions among pharmacists reflect these sectoral challenges. Alarming proportions of pharmacists intend to leave their current sectors, with 61.2% of hospital pharmacists and 55.8% of community pharmacists expressing such plans. The primary drivers are burnout and poor work–life balance [3,15,16]. Interestingly, pharmacists working in specialized hospital settings, such as oncology and critical care, report higher retention intentions driven by advanced practice opportunities and stronger interdisciplinary collaboration [4]. While offering research autonomy, academia faces moderate turnover rates (34.7%), often influenced by teaching loads and limited promotion pathways [15].

Despite the growing body of research on pharmacist well-being and career decisions in Saudi Arabia, most studies have been limited to a specific pharmacy workforce setting, leaving important gaps regarding cross-sector comparisons, sector mobility patterns, and the workforce impact of ongoing healthcare reforms [3,7,15]. In particular, little is known about how privatization efforts and sector transitions affect pharmacists’ well-being and career trajectories. Therefore, this study aims to explore the extent and determinants of job satisfaction, burnout, well-being, and career ambition among pharmacists practicing across different sectors in Saudi Arabia. By providing a comprehensive and cross-sector analysis, this study seeks to offer valuable insights that could inform workforce development strategies, support pharmacist retention. Accordingly, this study is designed as a descriptive, exploratory cross-sectional workforce survey and does not aim to establish causal relationships between healthcare reforms and individual career transitions.

## 2. Materials and Methods

This cross-sectional study used an electronic self-administered questionnaire distributed between December 2024 and January 2025 to pharmacists practicing in various sectors across Saudi Arabia. To achieve its objective, this study aimed to measure pharmacists’ well-being, job satisfaction, level of burnout, and career intentions in the hospital, community, pharmaceutical industry–related roles, and academic sectors. The survey was disseminated electronically by social media platforms to ensure broad participation from pharmacists working across various practice settings. This study adopts a descriptive, exploratory cross-sectional design to characterize pharmacists’ work experiences across sectors.

### 2.1. Study Population and Sampling

The target population of this study consisted of licensed pharmacists currently practicing across various sectors in Saudi Arabia. Eligible participants were pharmacists with a valid license, at least one year of professional experience, and who provided electronic informed consent. Pharmacists who were not currently practicing in Saudi Arabia, had less than one year of experience, or declined to participate were excluded from the study. According to recent estimates, approximately 30,000 licensed pharmacists are practicing in Saudi Arabia [15]. The minimum sample (n = 380) was calculated using a single-proportion formula (*p* = 0.50, 95% confidence, 5% margin of error). Participants were recruited using non-probability, convenience sampling via professional networks and social media, which may introduce selection bias and limit generalizability. Although the minimum required sample size was 380, all eligible responses received during the study period (n = 531) were included to enhance statistical power and sectoral representation. Saudi Arabia was categorized into five administrative regions (Central, Western, Eastern, Southern, and Northern), consistent with national statistical classifications. Eligibility was verified through mandatory screening questions at the beginning of the survey, including current practice in Saudi Arabia, licensure status, sector of employment, and years of professional experience. Responses not meeting eligibility criteria were excluded.

### 2.2. Survey Development and Measures

A structured, self-administered online questionnaire was developed specifically for this study to explore pharmacists’ work experiences, well-being, and career perspectives across different practice sectors in Saudi Arabia. The questionnaire was developed and administered using Google Forms (Google LLC, Mountain View, CA, USA). The survey was distributed through multiple professional online platforms, including LinkedIn, WhatsApp professional groups, and Telegram channels commonly used by pharmacists in Saudi Arabia. Due to the overlapping and open nature of these platforms, the sampling frame and response rate could not be determined, introducing potential selection bias. The survey included eight main sections: demographics, sector transition history, well-being and mental health, job satisfaction, burnout, workplace environment, career intentions, and professional development. The demographic section collected information on age, gender, nationality, region, marital status, income, years of experience, educational level, employment sector, and commuting time, while sector transition items explored the number and nature of movements between pharmacy sectors to understand patterns of career mobility (movement from hospital pharmacy to community pharmacy, academia, or another pharmacy-related sector). For the purpose of this study, Pharmaceutical industry–related roles included pharmacists working in pharmaceutical companies across roles such as medical affairs, pharmacovigilance, regulatory affairs, quality assurance and quality control, research and development, and medical representation. The core sections assessing well-being, job satisfaction, burnout, workplace environment, and career intentions were developed and validated by the research team through expert review and pilot testing to ensure content accuracy, internal consistency, and contextual relevance to pharmacy practice in Saudi Arabia. Content validity was established through expert evaluation by academic pharmacists specializing in clinical pharmacy and behavioral sciences. Their feedback was used to improve the structure and wording. Subsequently, a pilot study involving 20 pharmacists was conducted to assess clarity, reliability, and time to completion. The final version incorporated feedback from both expert reviewers and pilot participants and was distributed electronically over a four-week period using a secure online platform. Participants were recruited through professional networks, institutional channels, and social media to ensure wide representation across different pharmacy sectors. Well-being and mental health were assessed using a six-item scale addressing motivation, work–life balance, sense of purpose, emotional and mental health, stress levels, and organizational support for well-being, with responses rated on a 5-point Likert scale (1 = strongly disagree, 5 = strongly agree) yielding total scores from 6 to 30, where higher scores indicated better well-being (Cronbach’s α = 0.79). Job satisfaction was measured through an eight-item self-developed scale evaluating salary and incentives, professional growth, work–life balance, recognition, management support, work environment, job security, and autonomy, using the same Likert scale with total scores ranging from 8 to 40, where higher scores reflected greater satisfaction (Cronbach’s α = 0.83). Burnout was assessed using six items covering emotional exhaustion, detachment, workload, loss of motivation, work–life interference, and lack of fulfillment, with higher total scores (6–30) indicating greater burnout symptoms (Cronbach’s α = 0.75). The workplace environment was evaluated using a six-item scale assessing teamwork and collaboration, sense of safety and support, organizational appreciation of feedback, workload distribution, encouragement of professional growth, and opportunities for skill development, with higher scores (6–30) reflecting a more positive and supportive work environment (Cronbach’s α = 0.81). The final sections on career intentions and professional development explored participants’ plans to remain in or leave their current sector, preferred future sectors, reasons for career transitions such as financial incentives, work–life balance, or growth opportunities, and engagement in continuing education and professional development activities. All scales demonstrated acceptable to good internal consistency and were deemed appropriate for evaluating the experiences and perceptions of pharmacists across Saudi Arabia’s diverse practice settings. The questionnaire was designed to assess perceived workplace experiences rather than to provide clinical or diagnostic assessments. Accordingly, it should be interpreted as an exploratory, context-specific instrument. While validated instruments exist for burnout and mental health, concise context-specific measures were used to minimize respondent burden and enhance completion rates in a national workforce survey. Measurement limitations are acknowledged. The use of multiple digital platforms for questionnaire distribution may have introduced selection bias by preferentially reaching pharmacists who are more active on professional networks. Additionally, distributing the survey through several channels carries a potential risk of duplicate responses, which could affect the precision and strength of the results. These factors should be considered when interpreting the study findings. To mitigate this risk, screening questions were incorporated to ensure eligibility, and data cleaning procedures were applied to identify and review incomplete or potentially duplicate responses before analysis.

### 2.3. Statistical Analysis

Data were analyzed using IBM SPSS Statistics version 20.0 (IBM Corp., Armonk, NY, USA) after initial coding and cleaning in Microsoft Excel. Descriptive statistics were used to summarize the data, with categorical variables presented as frequencies and percentages and continuous variables expressed as medians with interquartile ranges (IQRs). Normality of continuous variables was assessed using the Shapiro–Wilk test. Accordingly, non-parametric statistical tests were applied where appropriate. The chi-square test was used for comparisons of categorical variables, with Fisher’s exact test and Monte Carlo correction applied when indicated. The Mann–Whitney U test and Kruskal–Wallis test were used to compare quantitative variables between groups. Correlations were assessed using Spearman’s rank correlation coefficient. All statistical tests were two-tailed, and a *p*-value ≤ 0.05 was considered statistically significant. Results were summarized and presented in tables and figures for clarity. Given the exploratory nature of the study, analyses were intended to describe associations rather than infer causality.

### 2.4. Ethical Considerations

Ethical clearance for the study was obtained from the Biomedical Research Ethics Committee, Umm Al-Qura University (Approval No.: HAPO-02-K-012-2024-11-2342). Electronic online consent was obtained from all participants before they started filling out the questionnaire. They were also informed about the study’s objective, that their participation was entirely voluntary, and that they had the right to withdraw at any time. Data confidentiality was ensured by collecting data anonymously and storing it securely with restricted access by the research team.

## 3. Results

### 3.1. Socio-Demographic Characteristics

A total of 531 pharmacists completed the survey. Table 1 summarizes the demographic characteristics of the participants studied. Most participants (61.2%) were in the 25–34-year-old age group, while 28.1% were 35–44 years old. Of the participants, 345 (65.0%) were males and 35% were females, yielding a male-to-female ratio of 1.85:1. Most participants (89.3%) were Saudi nationals. In terms of geographic distribution, 40.9% of participants lived in the Central Province, 28.8% in the Western Province, 14.9% in the Southern Province, 12.1% in the Eastern Province, and 3.4% were from the Northern Province. Additional workplace and background characteristics are presented in Table S1 (Supplementary Materials).

### 3.2. Workplace Data

Regarding monthly household income, approximately half of the participants (49.9%) had an income of 15,001–30,000 SAR, while 30.5% reported family income between 6000 and –15,000 SAR, 8.1% had income between 30,001 and 50,000 SAR, and 2.3% earned more than 50,000 SAR. According to working experience in pharmacy, 34.1% of participants had experience between 1 and 5 years, and 27.5% had experience between 6 and 10 years. Currently, the most common employment sector is pharmaceutical industry–related roles (29.4%), followed by clinical pharmacy (22.4%) and hospital pharmacy (20.2%).

Regarding educational background, approximately a third of participants (32.2%) held a Doctor of Pharmacy (PharmD) degree, 28.1% of them held a Master of Pharmacy degree, while 22.8% held a Bachelor of Pharmacy degree (BSc Pharm). Regarding commuting time, 32.8% of participants reported that it takes 31–60 min to drive from home to the workplace, and 32.2% of them said it takes 15–30 min. Furthermore, over half of the participants (61.4%) reported that they did not engage in remote work.

### 3.3. Sector Transition

The study reports that 162 participants (30.5%) changed their sector of employment, while 369 (69.5%) did not. Among those who changed sectors, 106 participants did so once, 37 did so twice, 12 did so three times, and 7 did so more than three times.

It was found that 22.6% of participants plan to stay in their current sector for 3–5 years, and 22.6% of participants for more than 5 years. The most frequent reasons were seeking a better work–life balance and better income (41.1%), followed by limited career advancement opportunities (20.9%), and burnout or stress (13.2%). Both the Research and Development career path and Clinical Pharmacy were considered as the dream path for 16.6% of participants each. The most frequent reason for choosing the dream path was the high professional growth and development potential. Less than half of the participants (48.9%) considered transitioning to another pharmacy sector. Further details regarding sector transition frequency and patterns are provided in Tables S2 and S3 (Supplementary Materials).

### 3.4. Well-Being and Mental Health

The median total well-being and mental health score was 20 (IQR: 17–23). Across individual items, the highest median response was observed for “My job provides a sense of purpose” (median 4, IQR 3–5), whereas workplace support for mental well-being showed a lower median (median 3, IQR 2–4).

### 3.5. Job Satisfaction

The median total job satisfaction score was 26 (IQR: 21–31). The highest median item response related to job security and stability and support from colleagues/management (median 4, IQR 3–4), while salary and growth opportunities showed comparatively lower median ratings (median 3, IQR 2–4).

### 3.6. Burnout

Participants reported burnout-related symptoms, with a median total burnout score of 16 (IQR: 12–20). Emotional exhaustion and perceived workload had higher median responses (median 3, IQR 2–4), while disconnection from colleagues/patients and lack of fulfillment showed lower median responses (median 2, IQR 2–3).

### 3.7. Workplace Environment

The median total workplace environment score was 20 (IQR: 15–24). Teamwork and collaboration showed the highest median rating (median 4, IQR 3–4), whereas opportunities for skill development showed a comparatively lower median (median 3, IQR 2–4). The full item-level distributions for well-being, job satisfaction, burnout, and workplace environment are detailed in Tables S4–S7 (Supplementary Materials).

### 3.8. Professional Development

Professional development refers to voluntary educational or training activities beyond mandatory CME requirements set by the Saudi Commission for Health Specialties. 337 (63.5%) of participants participated in professional development activities last year. Out of those 337 participants, the level of satisfaction with the availability of professional development resources was about 50.1% as satisfied and very satisfied. Workshops or conferences were the most common type of professional development activities (57.6%), followed by online courses or webinars (55.5%), then certification programs (52.2%). Research and academic publications were the least active (27.9%). More than half of the participants (59.9%) thought that professional development for their career growth is very important. Detailed professional development participation patterns are shown in Table S8 (Supplementary Materials).

### 3.9. Gender-Based Differences in Employment and Work Environment

There was a significant difference between males and females regarding the sector of current employment (*p* < 0.001), as females were more frequently represented in community and hospital pharmacy as shown in Table 2. In addition, there was a significant difference between males and females regarding sector change (*p* = 0.012), as it was significantly higher in males than in females. The Work Environment score was significantly higher in males than in females (*p* = 0.028). Participation in professional development activities in the past year was significantly higher in males than in females (*p* = 0.037). 

### 3.10. Age-Related Differences in Employment and Work Environment

There were statistically significant differences across age groups in several professional variables as shown in Table 3. The sector of current employment varied notably by age (*p* < 0.001); younger participants (20–24 years) were predominantly employed in community and pharmaceutical industry–related roles, whereas older participants (35–54 years) were more commonly engaged in clinical and academic roles. The frequency of sector changes also differed among age groups (*p* = 0.01), with mid-career pharmacists (25–44 years) reporting higher mobility between sectors. Furthermore, the intended duration of staying in the current sector was significantly associated with age (*p* < 0.001), as younger pharmacists were more likely to plan to leave within two years, while older participants tended to remain longer. In terms of work environment perceptions, the work environment score differed significantly by age group (*p* = 0.008), with younger pharmacists reporting more favorable perceptions compared to older counterparts. No significant differences were observed in burnout or job satisfaction scores across age groups. Detailed data are presented in Table 3. 

### 3.11. Differences Across Employment Sectors in Work Characteristics and Well-Being

There were significant differences across current employment sectors in several professional and psychological parameters. The frequency of sector changes (*p* < 0.001) and the number of times participants changed sectors (*p* < 0.001) varied significantly among sectors, with higher mobility observed among those working in pharmaceutical industry–related roles and regulatory, and administrative pharmacy compared to clinical or hospital pharmacists. Similarly, the intended duration of staying in the current sector was significantly associated with sector type (*p* < 0.001); participants in academia and clinical pharmacy reported a longer commitment to their current roles than those in community or industrial sectors. Marked differences were also found in the well-being and mental health scores (*p* < 0.001), job satisfaction scores (*p* < 0.001), and work environment scores (*p* < 0.001). Pharmacists in regulatory, administrative, and industrial sectors reported the highest well-being and job satisfaction, whereas those in community pharmacy reported the lowest. Although no significant variations were found in burnout levels (*p* = 0.328), participation in professional development activities differed slightly by sector (*p* = 0.040), with greater engagement among pharmacists in clinical and regulatory positions. Detailed cross-sector comparisons are presented in [Table 4].

### 3.12. Correlations Among Well-Being, Job Satisfaction, Work Environment, and Burnout

Well-being and mental health were positively correlated with job satisfaction (ρ = 0.718, *p* < 0.001) and work environment (ρ = 0.640, *p* < 0.001) and negatively correlated with burnout (ρ = −0.499, *p* < 0.001). Job satisfaction was strongly and positively associated with work environment (ρ = 0.763, *p* < 0.001) and negatively associated with burnout (ρ = −0.529, *p* < 0.001). Work environment was also negatively correlated with burnout (ρ = −0.512, *p* < 0.001). These findings indicate that higher well-being, satisfaction, and supportive work environments are associated with lower burnout. A detailed correlation matrix is provided in Table S9 (Supplementary Materials).

## 4. Discussion

This national study provides valuable insights into the experiences of pharmacists across multiple sectors in Saudi Arabia, exploring gender distribution, career mobility, burnout, well-being, job satisfaction, and within the context of ongoing healthcare transformation. Gender distribution patterns revealed notable disparities across pharmacy sectors. Female pharmacists were more commonly found in clinical and hospital settings, while male pharmacists dominated community and industrial sectors. These findings are consistent with previous literature indicating that cultural and workplace factors may influence sector distribution and career patterns among female pharmacists in Saudi Arabia [2,6,7]. Although recent policies aim to support greater female participation, particularly in the pharmaceutical industry and regulatory roles, structural and cultural barriers remain evident [5,12]. Interestingly, academia presented an inverse trend, with male pharmacists surpassing females in representation, contrasting with previous research that indicated balanced female involvement in academic pharmacy roles [13]. These evolving patterns highlight the importance of promoting entry opportunities for female pharmacists and ensuring supportive environments that foster their retention, progression, and leadership across all sectors [2,6,10]. The findings also demonstrated a dynamic pattern of sector mobility within the pharmacy workforce. Approximately one-third of participants had transitioned between sectors during their careers, reflecting both local and international workforce trends [16,17]. Sector mobility was ubiquitous among pharmacists working in regulatory, industrial, and community settings, while clinical pharmacy appeared more stable. The leading motivations for sector changes included better work–life balance, financial incentives, and limited opportunities for career advancement—factors that align with prior workforce studies in Saudi Arabia and globally [7,8,13]. Notably, male pharmacists reported higher rates of sector mobility than females, reinforcing previous evidence that women may face additional barriers or constraints when considering career changes [6]. Importantly, pharmacists who experienced sector changes were also more likely to participate in professional development activities, suggesting that sector mobility may be driven by a proactive approach to career growth and skill enhancement [13]. Burnout and well-being measures revealed important sectoral and demographic differences. Emotional exhaustion emerged as the most prominent component of burnout, particularly among community pharmacists, a finding consistent with earlier local studies [1,2]. The observed positive association between workload-related factors and burnout-related symptoms supports concerns regarding the demanding nature of pharmacy practice in specific settings [18]. Well-being scores were generally favorable; however, workplace support for mental health remained a key area of concern, aligning with prior observations of the need for stronger organizational interventions [10]. Gender analysis indicated that while overall burnout levels did not differ significantly, female pharmacists reported higher emotional exhaustion, consistent with previous research highlighting the need for gender-sensitive well-being strategies [6]. Participation in professional development was associated with lower reported burnout-related symptoms and better well-being outcomes [5]. Job satisfaction varied substantially across pharmacy sectors. Regulatory and hospital pharmacists reported higher satisfaction scores, while community pharmacists lagged behind. These findings depart from earlier research that positioned community pharmacy as a leading sector in satisfaction, suggesting that recent changes in work conditions may have altered satisfaction dynamics [2]. Organizational factors such as teamwork, managerial support, and workload management were strongly correlated with job satisfaction, underscoring their central role in shaping pharmacists’ work experiences [10]. Autonomy in decision-making and work–life balance were moderate across sectors but significantly better in clinical settings, reflecting the expanding scope of clinical pharmacy roles [5]. Notably, male pharmacists expressed greater satisfaction with their workplace environment, a finding that warrants further exploration to ensure gender equity in organizational practices [6].

Professional development engagement emerged as a critical factor influencing both job satisfaction and retention intentions. Participation rates in development activities exceeded previous local studies [2,13], indicating growing awareness of the importance of continuous learning in pharmacy practice. Workshops, conferences, and online courses were the most frequently utilized activities, with sectoral differences in resource satisfaction. Pharmacists in academic settings expressed the highest satisfaction, while community pharmacy pharmacists reported lower access to development opportunities. This disparity highlights the need to ensure equitable access to professional growth resources across all sectors [5,10]. Importantly, engagement in professional development was strongly correlated with both job satisfaction and long-term career retention intentions, emphasizing its central role in workforce sustainability [8]. Pharmacists working in settings perceived as more digitally advanced reported higher job satisfaction and better perceptions of the workplace environment. This finding aligns with recent literature emphasizing the positive influence of digital transformation on employee engagement and productivity [12]. Sectoral variation with industrial and regulatory sectors appearing more responsive to change than traditional community settings. These patterns highlight the need for targeted interventions to support sector-specific development and ensure that all pharmacists can benefit equally from the evolving healthcare landscape [7,13]. This study’s strengths include a large and diverse sample across multiple pharmacy sectors and regions, enhancing its representativeness and statistical power. However, this cross-sectional, self-reported survey cannot infer causality and is subject to recall and social desirability bias. The use of non-probability online recruitment may have introduced selection bias and limited representativeness. In addition, reliance on self-reported measures and the use of non-validated instruments may have resulted in measurement bias. The study did not capture the exact time interval over which employment sector changes occurred, limiting the ability to distinguish rapid workforce turnover from gradual career transitions. Finally, the higher representation of pharmacists working in pharmaceutical industry–related roles likely reflects greater engagement of industry-based professionals in online networks rather than national workforce proportions. Efforts to address burnout, particularly within community pharmacy, may warrant consideration of improving workload management, enhancing mental health support, and fostering more supportive organizational cultures. Gender disparities in sector distribution and mobility suggest the potential value of mentorship programs and structured career advancement pathways for female pharmacists. Expanding access to professional development resources across all sectors may be important, given its strong association with job satisfaction and retention. Future research should adopt longitudinal and mixed methods approaches to capture evolving workforce dynamics and assess the long-term impact of healthcare transformation on pharmacists’ well-being and career trajectories.

## 5. Conclusions

This study highlights key workforce dynamics among pharmacists in Saudi Arabia across various sectors. Gender disparities and sector mobility patterns were evident, with female pharmacists facing potentially more barriers in specific roles. Burnout was highest in community pharmacy, while the regulatory and pharmaceutical industry–related roles showed better job satisfaction. Professional development was strongly linked to job satisfaction and retention. Work–life balance and income were the commonly reported drivers of sector shifts, especially among younger pharmacists.

## Figures and Tables

**Table 1:** Socio-demographic characteristics of the participants.

Items		Participants (N = 531)	
		N	%
Age	20–24 years	26	4.9%
	25–34 years	325	61.2%
	35–44 years	149	28.1%
	45–54 years	29	5.5%
	≥55 years	2	0.4%
Gender	Female	186	35.0%
	Male	345	65.0%
Nationality	Saudi	474	89.3%
	Non-Saudi	57	10.7%
Region	Central Province	217	40.9%
	Eastern Province	64	12.1%
	Northern Province	18	3.4%
	Southern Province	79	14.9%
	Western Province	153	28.8%
Marital status	Divorced	15	2.8%
	Married	273	51.4%
	Single	242	45.6%
	Widowed	1	0.2%

**Table 2:** Relation between gender and different parameters.

		Females		Males		Test Value	*p*-Value
		(N = 186)		(N = 345)			
		N	%	N	%		
Sector of Current Employment	Academia/Research	5	2.70%	24	7.00%	X^2^ = 31.513	<0.001 **
	Clinical Pharmacy	62	33.30%	57	16.50%		
	Community Pharmacy	18	9.70%	55	15.90%		
	Hospital Pharmacy	46	24.70%	61	17.70%		
	Pharmaceutical industry–related roles	41	22.00%	115	33.30%		
	Regulatory and Administrative Pharmacy	14	7.50%	33	9.60%		
Sector Change	No	142	76.30%	227	65.80%	X^2^ = 6.34	0.012 **
	Yes	44	23.70%	118	34.20%		
How many times have you changed sectors? (n = 162)	More than 3 times	2	1.10%	5	1.40%	X^2^ = 1.13	0.77
	Once	26	14.00%	80	23.20%		
	Three times	4	2.20%	8	2.30%		
	Twice	12	6.50%	25	7.20%		
How long do you plan to stay in your current sector?	1–2 years	28	15.10%	52	15.10%	X^2^ = 2.239	0.692
	3–5 years	44	23.70%	76	22.00%		
	I do not know	58	31.20%	104	30.10%		
	Less than 1 year	20	10.80%	29	8.40%		
	More than 5 years	36	19.40%	84	24.30%		
Total Well-Being and Mental Health	Median (IQR)	20 (16–23)		20 (17–23)		^Z^_MWU_ = 1.188	0.235
	Range	6–30		6–30			
Job Satisfaction score	Median (IQR)	26 (21–31)		26 (22–32)		^Z^_MWU_ = 1.770	0.077
	Range	12–40		12–40			
	Range	5–25		5–25			
Work Environment score	Median (IQR)	19 (13–23)		20 (16–24)		^Z^_MWU_ = 2.199	0.028 *
	Range	6–30		6–30			
Burnout score	Median (IQR)	16 (13–20)		16 (12–20)		^Z^_MWU_ = 0.875	0.382
	Range	6–30		6–30			
Have you participated in professional development activities in the past year?	No	79	42.50%	115	33.30%	X^2^ = 4.354	0.037 *
	Yes	107	57.50%	230	66.70%		

*p* value > 0.05 is insignificant, * *p* value ≤ 0.05 is significant, ** *p* ˂ 0.01 is highly significant. IQR: Interquartile range; X^2^: Chi-square test; Z_MWU_: Mann–Whitney U test.

**Table 3:** Relation between age groups and different parameters.

		20–24 Years (N = 26)		25–34 Years (N = 325)		35–44 Years (N = 149)		45–54 Years (N = 29)		55+ Years (N = 2)		*p*-Value
		N	%	N	%	N	%	N	%	N	%	
Sector of Current Employment	Academia/Research	1	3.8%	7	2.2%	18	12.1%	3	10.3%	0	0.0%	<0.001 **
	Clinical Pharmacy	0	0.0%	53	16.3%	47	31.5%	18	62.1%	1	50.0%	
	Community Pharmacy	10	38.5%	43	13.2%	18	12.1%	2	6.9%	0	0.0%	
	Hospital Pharmacy	3	11.5%	67	20.6%	34	22.8%	2	6.9%	1	50.0%	
	Pharmaceutical industry–related roles	10	38.5%	128	39.4%	15	10.1%	3	10.3%	0	0.0%	
	Regulatory and Administrative Pharmacy	2	7.7%	27	8.3%	17	11.4%	1	3.4%	0	0.0%	
Sector Change	No	20	76.9%	218	67.1%	106	71.1%	24	82.8%	1	50.0%	0.302 ^FE^
	Yes	6	23.1%	107	32.9%	43	28.9%	5	17.2%	1	50.0%	
How many times have you changed sectors?	No	20	76.9%	218	67.1%	106	71.1%	24	82.8%	1	50.0%	0.038 ** ^FE^
	Once	6	23.1%	76	23.4%	22	14.8%	2	6.9%	0	0.0%	
	Twice	0	0.0%	20	6.2%	15	10.1%	2	6.9%	0	0.0%	
	Three times	0	0.0%	8	2.5%	3	2.0%	1	3.4%	0	0.0%	
	More than 3 times	0	0.0%	3	0.9%	3	2.0%	0	0.0%	1	50.0%	
How long do you plan to stay in your current sector?	1–2 years	11	42.3%	57	17.5%	12	8.1%	0	0.0%	0	0.0%	<0.001 ** ^FE^
	3–5 years	3	11.5%	74	22.8%	34	22.8%	9	31.0%	0	0.0%	
	I do not know	5	19.2%	86	26.5%	61	40.9%	9	31.0%	1	50.0%	
	Less than 1 year	4	15.4%	34	10.5%	7	4.7%	4	13.8%	0	0.0%	
	More than 5 years	3	11.5%	74	22.8%	35	23.5%	7	24.1%	1	50.0%	
Well-Being and Mental Health score	Median (IQR)	20 (18–23.5)		20 (17–24)		20 (17–22)		22 (18–23)		22 (20.5–23.5)		0.848
	Range	6–29		6–30		6–30		9–28		19–25		
Job Satisfaction score	Median (IQR)	26 (17.25–30.8)		27 (22–32)		25 (20–30)		26 (21–30)		29 (27.5–30.5)		0.135
	Range	9–40		8–40		8–40		9–40		26–32		
	Range	6–23		5–25		5–25		5–25		10–15		
Work Environment score	Median (IQR)	22.5 (17.25–26.8)		21 (16–25)		19 (13–23)		18 (14–23)		21.5 (19.75–23.3)		0.008 **
	Range	8–30		6–30		6–30		6–28		18–25		
Burnout score	Median (IQR)	14.5 (12–18)		16 (12–20)		16 (12–21)		16 (11–21)		16 (15–17)		0.969
	Range	7–28		6–30		7–30		6–30		14–18		
Have you participated in professional development activities in the past year?	No	13	50.0%	108	33.2%	57	38.3%	15	51.7%	1	50.0%	0.109 ^FE^
	Yes	13	50.0%	217	66.8%	92	61.7%	14	48.3%	1	50.0%	

*p* value > 0.05 is insignificant; ** *p* ˂ 0.01 is highly significant; IQR: Interquartile range; ^FE^: Fisher’s exact test.

**Table 4:** The Relationship between the current employment sector and different parameters.

		Academia/Research (N = 29)		Clinical Pharmacy (N = 119)		Community Pharmacy (N = 73)		Hospital Pharmacy (N = 107)		Pharmaceutical Industry–Related Roles (N = 156)		Regulatory and Administrative Pharmacy (N = 47)		*p*-Value
		N	%	N	%	N	%	N	%	N	%	N	%	
Sector change	No	16	55.2%	107	89.9%	50	68.5%	86	80.4%	89	57.1%	21	44.7%	<0.001 **
	Yes	13	44.8%	12	10.1%	23	31.5%	21	19.6%	67	42.9%	26	55.3%	
How many times have you changed sectors?	No	16	55.2%	107	89.9%	50	68.5%	86	80.4%	89	57.1%	21	44.7%	<0.001 ** ^FE^
	More than 3 times	0	0.0%	1	0.8%	1	1.4%	1	0.9%	1	0.6%	3	6.4%	
	Once	9	31.0%	5	4.2%	13	17.8%	13	12.1%	51	32.7%	15	31.9%	
	Three times	1	3.4%	1	0.8%	1	1.4%	1	0.9%	6	3.8%	2	4.3%	
	Twice	3	10.3%	5	4.2%	8	11.0%	6	5.6%	9	5.8%	6	12.8%	
How long do you plan to stay in your current sector?	1–2 years	4	13.8%	8	6.7%	23	31.5%	13	12.1%	27	17.3%	5	10.6%	<0.001 **
	3–5 years	9	31.0%	30	25.2%	9	12.3%	20	18.7%	38	24.4%	14	29.8%	
	I do not know	13	44.8%	49	41.2%	17	23.3%	34	31.8%	36	23.1%	13	27.7%	
	Less than 1 year	1	3.4%	6	5.0%	13	17.8%	16	15.0%	9	5.8%	4	8.5%	
	More than 5 years	2	6.9%	26	21.8%	11	15.1%	24	22.4%	46	29.5%	11	23.4%	
Well-Being and Mental Health score	Median (IQR)	21 (18–23)		20 (17–23)		18 (15–22)		21 (17–24)		22 (19–25)		23 (19–25)		<0.001 **
	Range	12–28		8–30		6–30		7–30		19–25		10–30		
Job Satisfaction score	Median (IQR)	27 (22–29)		26 (21–30)		23 (17–27)		29 (24–32.5)		29 (26–32)		30 (24–34)		<0.001 **
	Range	12–40		8–40		8–40		8–40		26–32		9–40		
	Range	5–22		5–25		5–25		5–25		10–15		6–23		
Work Environment score	Median (IQR)	19 (13–23)		18 (14–23)		18 (14–23)		22 (18–26)		21.5 (18–25)		23 (16–27)		<0.001 **
	Range	6–30		6–30		6–30		6–30		18–25		6–30		
Burnout score	Median (IQR)	16 (12–19)		16 (12–21)		17 (13–21)		16 (12–20)		16 (14–18)		15 (11–20)		0.328
	Range	7–27		6–30		7–30		6–30		14–18		7–28		
Have you participated in professional development activities in the past year?	No	12	41.4%	33	27.7%	32	43.8%	49	45.8%	55	35.3%	13	27.7%	0.040 *
	Yes	17	58.6%	86	72.3%	41	56.2%	58	54.2%	101	64.7%	34	72.3%	

*p* value > 0.05 is not significant, * *p* value ≤ 0.05 is significant, ** *p* ˂ 0.01 is highly significant. IQR: Interquartile range; analysis carried out by Chi-Square test, ^FE^: Fischer’s exact test.

## Data Availability

The data presented in this study are available on request from the corresponding author due to privacy and ethical reasons.

## Supplementary Materials

The following supporting information can be downloaded at: https://www.mdpi.com/article/10.3390/pharmacy14010018/s1. Tables S1: Working data among the studied participants; Table S2: Distribution of sector change among the participants studied; Table S3: Participants’ Perspectives on Sector Preferences, Transition Plans, and Motivations; Table S4: Distribution of the participants studied regarding Well-Being and Mental Health data; Table S5: Distribution of the participants regarding Job Satisfaction; Table S6: Distribution of the studied participants regarding burnout questions; Table S7: Distribution of the studied participants regarding workplace environment; Table S8: Professional development data among the participants studied; Table S9: Spearman correlations among Well-Being, Job Satisfaction, Work Environment, and Burnout (N = 531).
