# Supplementary material for: Pharmacists’ Work Experiences and Career Dynamics in Saudi Arabia: A Cross-Sector Study
Source: Pharmacy (Basel). 2026 Jan 27;14(1):18. doi: 10.3390/pharmacy14010018 (PMC12922034; doi:10.3390/pharmacy14010018)
Supplement: Supplementary file 1 [file pharmacy-14-00018-s001.zip › pharmacy-3986129-supplementary.pdf]

Supplementary Tables

Table S1: Working data among the studied participants.

	Participants (N= 531)	
	N	%
**Monthly Household Income**		
15,001 - 30,000 SAR	265	49.9%
30,001 - 50,000 SAR	43	8.1%
6,000 - 15,000 SAR	162	30.5%
Less than 6,000 SAR	17	3.2%
More than 50,000 SAR	12	2.3%
Prefer not to say	32	6.0%
**Years of Experience in Pharmacy**		
1–5 years	181	34.1%
11–15 years	76	14.3%
6–10 years	146	27.5%
Less than 1 year	45	8.5%
More than 15 years	83	15.6%
**Sector of Current Employment**		
Academia/Research	29	5.5%
Clinical Pharmacy	119	22.4%
Community Pharmacy	73	13.7%
Hospital Pharmacy	107	20.2%
Pharmaceutical industry related roles	156	29.4%
Regulatory and Administrative Pharmacy	47	8.9%
**Educational qualifications**		
Bachelor of Pharmacy (BSc Pharm)	121	22.8%

	Participants (N= 531)	
	N	%
Doctor of Pharmacy (PharmD)	171	32.2%
Master's Degree	149	28.1%
PhD	26	4.9%
Postgraduate Diploma or Residency	63	11.9%
Intermediate University Degree in Health Sciences in Pharmacy	1	0.2%
**How long does it take you to drive from your home to your workplace?**		
15–30 minutes	171	32.2%
31–60 minutes	174	32.8%
I work remotely or from home	6	1.1%
Less than 15 minutes	101	19.0%
More than 60 minutes	70	13.2%
Not mentioned	9	1.7%
**How many days do you work remotely in a year at your workplace?**		
1–10 days	84	15.8%
11–30 days	57	10.7%
31–60 days	22	4.1%
More than 60 days	33	6.2%
None (I do not work remotely)	326	61.4%
Not mentioned	9	1.7%

Table S2: Distribution of sector change among the participants studied.

	Participants (N= 531)	
	N	%
**Have you changed your sector of employment (your professional setting)?**		
**No (Never changed sector)**	369	69.5%
**Yes**	162	30.5%
• **Once**	106	20.0%
• **Twice**	37	7.0%
• **Three times**	12	2.3%
• **More than 3 times**	7	1.3%

Table S3: Participants' Perspectives on Sector Preferences, Transition Plans, and Motivations.

Items		Participants (N= 531)	
		N	%
**1. How long do you plan to stay in your current sector?**	Less than 1 year	49	9.2%
	1–2 years	80	15.1%
	3–5 years	120	22.6%
	More than 5 years	120	22.6%
	I do not know	162	30.5%
**2. If you plan to leave, what is your primary reason?**	Burnout or stress	70	13.2%
	Pursuing a different health profession	69	13.0%
	Transitioning to a non-health field	53	10.0%
	Seeking better work-life balance and better income	218	41.1%
	Limited career advancement opportunities	111	20.9%
	No, I plan to stay in my current role	10	1.9%

Items		Participants (N= 531)	
		N	%
**3. What is your dream career path?**	Research and Development	88	16.6%
	Clinical Pharmacy	88	16.6%
	Pharmaceutical Companies	81	15.3%
	Regulatory Affairs	81	15.3%
	Academia	62	11.7%
	Pharmaceutical industry related roles	56	10.5%
	Hospital/Healthcare	48	9.0%
	Community Pharmacy	9	1.7%
	Others as own Outside Pharmacy sector, own business or not knowing yet	15	2.8%
	Any sector	3	0.6%
**4. Why is this your dream sector? ***	Passion for helping patients or the community	132	24.9%
	Interest in research, innovation, or drug development	141	26.6%
	Desire to work in a leadership or administrative role	158	29.8%
	Opportunity to contribute to education and training	142	26.7%
	High potential for professional growth and development	203	38.2%
	Preference for a collaborative and team-oriented environment	52	9.8%
	Financial stability or income potential	146	27.5%
	Flexibility and work-life balance	118	22.2%
	Personal interest or long-term ambition	86	16.2%
**5. Have you considered transitioning to another pharmacy sector?**	No	273	51.4%
	Yes	258	48.6%

*More than one option was selected

Table S4: Distribution of the participants studied regarding Well-Being and Mental Health data.

	Participants (N= 531)				
	Median	IQR		Range	
1. I feel motivated and energized in my daily tasks.	3.0	3.0	4.0	1.0	5.0
2. I have a sufficient work-life balance.	3.0	2.0	4.0	1.0	5.0
3. My job provides a sense of purpose.	4.0	3.0	5.0	1.0	5.0
4. I feel mentally and emotionally healthy.	3.0	3.0	4.0	1.0	5.0
5. I often experience high-stress levels that impact my productivity.	4.0	3.0	4.0	1.0	5.0
6. My workplace actively supports my mental well-being.	3.0	2.0	4.0	1.0	5.0
Total Well-Being and Mental Health	20.0	17.0	23.0	6.0	30.0

IQR: Interquartile range

Table S5: Distribution of the participants regarding Job Satisfaction.

	Participants (N= 531)				
	Median	IQR		Range	
1. Salary and financial incentives	3.0	2.0	4.0	1.0	5.0
2. Opportunities for professional growth and career advancement	3.0	2.0	4.0	1.0	5.0

**3. Work-life balance and scheduling flexibility**	3.0	2.0	4.0	1.0	5.0
**4. Recognition and appreciation for contributions**	3.0	2.0	4.0	1.0	5.0
**5. Support from colleagues and management**	4.0	3.0	4.0	1.0	5.0
**6. Physical and organizational work environment**	3.0	2.0	4.0	1.0	5.0
**7. Job security and stability**	4.0	3.0	4.0	1.0	5.0
**8. Degree of autonomy in decision-making**	3.0	3.0	4.0	1.0	5.0
**Total Job Satisfaction**	26.0	21.0	31.0	8.0	40.0

IQR: Interquartile range

Table S6 : Distribution of the studied participants regarding burnout questions.

	Participants (N= 531)				
	**Median**	**IQR**		**Range**	
**1. I feel emotionally exhausted at the end of my workday**	3.0	2.0	4.0	1.0	5.0
**2. I feel disconnected from my colleagues and/or patients.**	2.0	2.0	3.0	1.0	5.0
**3. My workload feels overwhelming.**	3.0	2.0	4.0	1.0	5.0
**4. I feel unmotivated or disinterested in my job.**	3.0	2.0	3.0	1.0	5.0
**5. My work negatively impacts my personal life.**	2.0	2.0	4.0	1.0	5.0
**6. I feel a lack of achievement or fulfillment in my role.**	2.0	1.0	3.0	1.0	5.0
**Total burnout score**	16.0	12.0	20.0	6.0	30.0

IQR: Interquartile range

Table S7: Distribution of the studied regarding workplace environment.

	Participants (N= 531)				
	Median	IQR		Range	
**My workplace promotes teamwork and collaboration.**	4.0	3.0	4.0	1.0	5.0
**I feel safe and supported at my workplace.**	3.0	3.0	4.0	1.0	5.0
**My organization values employees, feedback, and suggestions.**	3.0	2.0	4.0	1.0	5.0
**My workload is manageable and well-distributed.**	3.0	2.0	4.0	1.0	5.0
**My organizational culture encourages professional growth and innovation.**	3.0	2.0	4.0	1.0	5.0
**My workplace provides clear opportunities for skill development.**	3.0	2.0	4.0	1.0	5.0
**Total Workplace environment**	20.0	15.0	24.0	6.0	30.0

IQR: Interquartile range

Table S8: Professional development data among the participants studied.

	Participants (N= 531)	
	**N**	**%**
**Have you participated in professional development activities in the past year?**		
No	194	36.5%
Yes	337	63.5%
**Rate your satisfaction with the availability of professional development resources (n=337)**		
Very dissatisfied	13	3.9%
Dissatisfied	49	14.5%
Neutral	106	31.5%
Satisfied	111	32.9%
Very satisfied	58	17.2%
**What types of professional development activities have you engaged in (n=337)***		
Workshops or conferences	194	57.6%
Online courses or webinars	187	55.5%
Certification programs	176	52.2%
Mentorship opportunities	92	27.3%
Research and academic publications	94	27.9%
**How important is professional development for your career growth (n=337)**		
Not important at all	6	1.8%
Not important	14	4.2%
Neutral	45	13.4%
Important	70	20.8%
Very important	202	59.9%

**The responder may have one or more responses to this question.*

Table S9: Spearman correlations among Well-Being, Job Satisfaction, Work Environment, and Burnout (N = 531).

Variables	Well-Being & Mental Health	Job Satisfaction	Work Environment	Burnout
**Well-Being & Mental Health**	—	0.718**	0.640**	–0.499**
**Job Satisfaction**	0.718**	—	0.763**	–0.529**
**Work Environment**	0.640**	0.763**	—	–0.512**
**Burnout**	–0.499**	–0.529**	–0.512**	—

Note: Spearman's rho (two-tailed). $p < 0.001$ for all non-diagonal coefficients. Diagonal (self-correlations) omitted (=1.000).
